# Associations between frailty syndrome and sociodemographic characteristics in long-lived individuals of a community**^1^**

**DOI:** 10.1590/1518-8345.1770.2886

**Published:** 2017-06-05

**Authors:** Clóris Regina Blanski Grden, Maria Helena Lenardt, Jacy Aurelia Vieira de Sousa, Luciana Kusomota, Mara Solange Gomes Dellaroza, Susanne Elero Betiolli

**Affiliations:** 2 PhD, Adjunct Professor, Departamento de Enfermagem e Saúde Pública, Universidade Estadual de Ponta Grossa, Ponta Grossa, PR, Brazil.; 3 PhD, Professor, Departamento de Enfermagem, Universidade Federal do Paraná, Curitiba, PR, Brazil.; 4 PhD, Professor, Escola de Enfermagem de Ribeirão Preto, Universidade de São Paulo, WHO Collaborating Centre for Nursing Research Development, Ribeirão Preto, SP, Brazil.; 5 PhD, Adjunct Professor, Departamento de Enfermagem, Universidade Estadual de Londrina, Londrina, PR, Brazil.; 6 PhD, Professor, Sociedade Educacional Herrero, Curitiba, PR, Brazil.

**Keywords:** Elderly, Aged, 80 and Over, Frail Elderly, Geriatric Nursing, Socioeconomic Factors, Aging

## Abstract

**Objective:**

investigating the association between frailty syndrome and sociodemographic characteristics in long-lived individuals of a community.

**Method:**

a cross-sectional study with a proportional stratified sample consisting of 243 long-lived individuals. A structured instrument, scales and tests that comprise evaluating frailty were applied for data collection. Univariate and multivariate analyzes were performed by logistic regression (p<0.05) by Statistica 10^(r)^ software and Odds Ratio (95% Confidence Interval) were calculated for the predictive models.

**Results:**

of the 243 long-lived individuals evaluated, 36 (14.8%) were frail, 55 (63.8%) were pre-frail and 52 (21.4%) were not frail. A predominance of females (n=161; 66.3%), widows/widowers (n=158; 65%), who lived with family members (n=144; 59.3%) and in a self-reported satisfactory financial situation (n=108; 44.5%) was observed. A significant association was found between the demographic variable of age (p=0.043) and frailty syndrome. The best predictor model for the syndrome included the variables: gender, age and household companion.

**Conclusion:**

the variable of age contributed most to the fragilization process of long-lived individuals residing in the community. It is essential that gerontological nursing care contemplates early detection of this syndrome, considering age as being indicative of care needs.

## Introduction

The increase in the number of elderly people (also called long-lived individuals or oldest old) at 80 years or more is a world-wide reality. A decrease in physical reserves and an increase in vulnerability to internal and external stressors is common in this population segment, culminating in the development of physical frailty^1^. Researchers define it as a medical syndrome with multiple causes and related factors, characterized by a decrease in the body's homeostatic reserve capacity and resistance to stressors, which result in cumulative declines in multiple physiological systems causing vulnerability and adverse clinical outcomes^1^ ^-^ ^2^.

In order to evaluate the syndrome, the frailty/fragility phenotype stands out, being composed of markers that include: slow gait, reduced hand grip strength, unintentional weight loss, self-reported exhaustion and low level of physical activity. Long-lived individuals who do not have any of the markers are considered as not frail, those with one or two markers are pre-frail, and three or more characterize frail aged adults^2^.

The frequency of physical frailty presents great variability, whether in homogeneous or distinct long-lived populations^3^. In younger aged adults (60-69 years), values between 6.9% and 9.3%^2^ ^,^ ^4^ ^)^ are predominant, while the index is significantly higher for long-lived individuals (≥80 years), ranging from 16% to 26% ^(^ ^3^.

High prevalence rates of the syndrome in the national context do not correspond to the number of publications found in Brazilian gerontology nursing literature or in general health^5^. This suggests the need for further studies investigating the predictive characteristics of the syndrome, since they provide valuable subsidies for care management. These studies especially focused on long-lived individuals cannot be disregarded, as they are high risk for frailty with a higher probability of changing from pre-frail to frail^2^, and which predisposes this age group to hospitalizations, falls and dependencies.

Among the factors that determine frailty development, some sociodemographic factors stand out even though they are often ignored by the healthcare team providing care, and they should be systematically investigated in evaluating longed-lived older adults. In developing countries, higher values of frailty in elderly^6^ have been identified. In investigations carried out in Mexico^7^ and Peru^8^, researchers have demonstrated frailty associated with sociodemographic variables, including female gender^7^ and age^7^ ^-^ ^8^.

Despite sociodemographic variables that are associated with frailty being known, we highlight a lack of national studies that investigate predictive models of frailty based on these characteristics of the population. In view of the above, the objective of the present study was to investigate the association between frailty syndrome and sociodemographic characteristics of long-lived individuals in a community.

## Method

A cross-sectional study developed with long-lived individuals (≥80 years) living in a community of households in an area covered by three Basic Health Units (*UBS*) belonging to the Boa Vista Sanitary District, in the city of Curitiba, Paraná, Brazil. As criteria the choice of these *UBS* ^9^ ^)1^ was the representativeness of different social strata associated to the greater number of long-lived adults enrolled in the *UBS*. A proportional stratified sample was adopted, considering the number of long-lived individuals enrolled in each *UBS*, so that none of the *UBS* was over or underestimated. The sample calculation considered the population of long-lived individuals enrolled in the three *UBS* (N=503), an 80% (1-ß) beta value, a 5% significance level (α=0.05) and a 10% significant minimum difference between the proportions of long-lived individuals with frailty. The sample size was increased by 10% due to the possibilities of losses and refusals, which resulted in the final sample being 243 long-lived individuals.

The selection of the aged adults was randomly carried out through a draw based on the list of long-lived individuals registered at the *UBSs* generated by the city's electronic system. Home visits were made, and a new name was drawn in cases of refusal or absence (three attempts for each household).

Inclusion criteria were: a) being ≥80 years of age; b) being enrolled in one of the *UBS* participating in the study; c) scoring higher than the cutoff point in the Mini Mental State Examination (MMSE)^10^, which was 13 points for illiterates, 18 for having an average or low education level, and 26 points for having a high education level^11^. 

The family caregiver was invited to participate in cases where long-lived individuals did not have cognitive conditions (n=36) to answer the research questions, and for which the following inclusion criteria were considered: a) being ≥18 years of age; b) being a family caregiver; c) living with the aged adult for at least three months. Long-lived individuals who were physically unable to perform the physical tests (n=15) or in chemotherapeutic treatment (n=1) were excluded.

Data collection was carried out from January 2013 to September 2015 by previously trained scientific initiation fellowship, master's and doctorate students. A pilot study with ten long-lived individuals was conducted for verification and adequacy of the instrument. 

The structured questionnaire included sociodemographic variables of interest to the study adapted from the Brazilian Institute of Geography and Statistics^12^, and categorized according to statistical recommendation and/or studies that supported the methodology ^(^ ^11^: gender (female, male); age (≥80 and <87, ≥87 and <93, ≥93 and <100 years); marital status (widower, married, single); education level (illiterate, low, average, high); household situation (living alone, living with family members, living with a partner); self-reported financial situation (unsatisfactory, average, satisfactory); individual monthly income (insufficient ≤1.0 minimum wage (MW), average >1.0 MW and ≤2.0 MW, satisfactory >2 MW) and monthly family income (insufficient ≤2.0 MW, average >2.0 and ≤5.0 MW, satisfactory >5 MW).

Physical frailty markers were evaluated according to the authors' proposal^2^ ^)^ in the *Cardiovascular Health Study* (CHS), a prospective and observational reference study conducted in the United States with 5,317 community aged adults between 65 to 101 years of age. 

**Figure 1:**
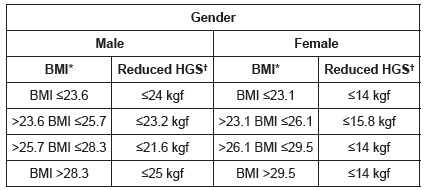
Cutoff points adopted by gender according to BMI classifications, which indicated reduced HGS in long-lived participants. Curitiba-PR, Brazil, 2015.

Hand Grip Strength (HGS) was measured with a Jamar(r) hydraulic dynamometer according to the *American Society of Hand Therapists* ^13^. The highest of three obtained measurements presented in kilograms (Kgf) was considered^14^, adjusted according to gender and body mass index^2^ (BMI=weight/height^2)^, and values in the lowest fifth were considered frailty markers, as observed in Figure 1. 

*Body mass index †Hand Grip Strength

In order to evaluate the Gait Speed (GS) marker, the long-lived participants were instructed to walk a distance of six meters^15^ in a usual manner on a flat surface marked by two marks of four meters distance from one another. Only the intermediate four-meter course was considered to reduce the effects of acceleration and deceleration. Time was measured in seconds by a digital stopwatch and divided by the four-meter trajectory, thus resulting in a GS in meters/second, as according to an international study^2^ ^).^

The results were adjusted according to gender and height^2^, and then divided into two categories based on the median (50th percentile): men ≤166 cm and women ≤152 cm (below or equal to the median); and men > 166 cm and women > 152 cm (above the median). The cutoff points were set in the lower fifth^2^ for each category with the following values for men and women, respectively: below or equal to the median of ≥9.65 s and ≥13.0 seconds; and above the median of ≥7.97 s and ≥11.6 seconds. Values equal to or greater than the cutoff points on the walking test were considered as markers for the syndrome.

Unintentional weight loss was verified according to participants' self-report regarding the following questions: a) Have you lost weight in the last twelve months? b) If so, how many kilos? The caregiver was consulted if they did not remember their weight a year ago. Unintentional (without diet or exercise) weight loss reported as greater than or equal to 4.5 kg in the last twelve months was considered a marker for physical frailty^2^.

Fatigue/exhaustion was evaluated based on self-reporting for a question from the Depression Scale of the Center of Epidemiological Studies^16^: "Do you feel full of energy?" A visual scale was used to measure energy level using a ruler numbered from zero to ten, with zero being the minimum energy level and ten the maximum. Negative responses provided by long-lived individuals to the question associated with an energy value equal to or less than three points on the ruler were considered as a marker for the syndrome^17^.

The reduction in physical activity level was evaluated using the "Physical Activity Level Questionnaire for the Aged" - *CuritibAtiva*. The validated instrument^18^ contains twenty questions subdivided into: systematic practice of physical activities (n=7); heavy household or occupational tasks (n=7); and social and leisure activities (n=6). The questions refer to the frequency and duration of the activities performed in the last week, and the score is converted into the following classification: inactive (0-32); slightly active (33-82); moderately active (83-108); active (109-133); very active (≥134). Classifications compatible with the inactive and slightly active groups were considered as a marker for the syndrome.

The results were tabulated and analyzed in *^_Statistica10(r)_^* software. Descriptive statistics were obtained through distribution of absolute and relative frequency, mean, standard deviation, mode and median. The association between frailty and independent variables was analyzed by the chi-square test, with *p*<0.05. Two groups were analyzed (Cluster analysis) in the multivariate model using logistic regression, which lead to merging the pre-frail and not frail categories. The priority response for prediction was defined as the frail response, and the other category (not frail) was attributed as its complement in order to follow a model associated with a binomial distribution. 

A complete predictive model was developed for frailty in which all variables of the study were included. Using the *Forward Stepwise* method, the sociodemographic variables were individually entered into multiple logistic regression analyzes, starting with those that showed a lower individual p-value for the complete model. The Likelihood Ratio Test (LRT) was implemented for selecting the models, and the quality of fit was assessed by *Deviance* analysis. The respective Odds Ratio (OR) and Confidence Interval (CI) of 95% were also calculated.

The study complied with national and international standards of research ethics involving human subjects, and was approved by the Research Ethics Committee in Human Beings of the institution under registration CEP/SD: 156.413.

## Results

For physical frailty, 36 (14.8%) of the long-lived individuals were classified as frail, 52 (21.4%) as not frail, and 155 (63.8%) as pre-frail. A predominance of females (n=161; 66.3%), in the age group ≥ 80 to <87 years (n=181; 74.5%), with a mean age of 84.4 (SD=3.8) was observed. The majority were widowed (n=158; 65%), with a low education level (n=137; 56.4%), and living with family members (n=144; 59.3%). Of the participants, 108 (44.5%) considered their financial situation satisfactory; however, most of the long-lived individuals (n=181; 74.5%) reported their individual monthly income as insufficient (receiving up to a minimum wage)^2*^. Regarding frail individuals (n=36; 14.8%), the majority were females (n=25; 69.4%), widowed (n=27; 75%), living with family members (n=28; 77.7%), with average financial situation (n=16; 44.4%) and individual and familiar monthly income classified as insufficient, in the same frequency (n=28; 77.8%). There was a significant association between age (*p=*0.0432) and frailty (Table 1).

**Table 1:** Association between frailty and sociodemographic characteristics in long-lived individuals by levels of frailty. Curitiba, PR, Brazil, 2015

Variable	Classification	Total (%)	Frail (%)	Pre-Frail (%)	Not Frail (%)	*p-value*
Gender	Female	161(66.3)	25(69.4)	103(66.5)	33(63.5)	0.8403
	Male	82(33.7)	11(30.6)	52(33.5)	19(36.5)	
Age	≥80 to <87 years	181(74.5)	24(66.7)	112(72.2)	45(86.5)	0.0432
	≥87 to <93 years	52(21.4)	8(22.2)	37(23.9)	7(13.5)	
	≥93 to <100 years	10(4.1)	4(11.1)	6(3.9)	0(0)	
Marital Status	Widowed	158(65)	27(75)	101(65.2)	30(57.7)	0.4173
	Married	73(30)	7(19.5)	48(31)	18(34.6)	
	Single	12(5)	2(5.5)	6(3.8)	4(7.7)	
Education Level	Illiterate	90(37)	12(33.3)	61(39.3)	17(32.7)	0.7514
	Low	137(56.4)	22(61.1)	82(52.9)	33(63.5)	
	Average	10(4.1)	1(2.8)	7(4.5)	2(3.8)	
	High	6(2.5)	1(2.8)	5(3.3)	0(0)	
Household (living situation)	Alone	65(26.7)	6(16.7)	41(26.5)	18(34.6)	0.1088
	With family members	144(59.3)	28(77.7)	89(57.4)	27(51.9)	
	With partner	34(14)	2(5.6)	25(16.1)	7(13.5)	
Financial situation	Unsatisfactory	47(19.3)	5(13.9)	33(21.3)	9(17.3)	0.7379
	Average	88(36.2)	16(44.4)	54(34.8)	18(34.6)	
	Satisfactory	108(44.5)	15(41.7)	68(43.9)	25(48.1)	
Individual Monthly Income	Insufficient	181(74.5)	28(77.8)	117(75.5)	36(69.2)	0.7422
	Average	51(21)	7(19.4)	32(20.6)	12(23.1)	
	High	11(4.5)	1(2.8)	6(3.9)	4(7.7)	
Monthly Family Income	Insufficient	182(74.9)	28(77.8)	114(73.5)	40(76.9)	0.5328
	Average	54(22.2)	7(19.4)	38(24.6)	9(17.3)	
	High	7(2.9)	1(2.8)	3(1.9)	3(5.8)	
Total		243(100)	36(14.8)	155(63.8)	52(21.4)	

Three predictive logistic models of frailty for long-lived individuals were carried out. The complete model (*p*=0.352) included the variables of gender, age, marital status, household situation, education level, financial situation, individual and family income. Model 1 (*p*=0.075) included gender, age and household situation. Model 2 (*p*=0.045) considered the variables age, household situation and individual income. No significant associations were found between the models (Table 2).

**Table 2:** Predictive models for frailty in long-lived individuals, according to sociodemographic variables. Curitiba, PR, Brazil, 2015

Variables		Complete Model OR (95% CI) ***p*** =0.3527	***p***	Model 1 OR (95% CI) ***p*** =0.074	***p***	Model 2 OR (95% CI) ***p*** =0.045	***p***
Gender		0.90 (0.36-2.28)	0.836	1.19 (0.54-2.67)	0.66		
Age							
	≥80 to <87 years	0.26 (0.06-1.07)	0.062	0.28 (0.07-1.10)	0.068	0.28 (0.07-1.10)	0.068
	≥87 to <93 years	0.25 (0.06-1.20)	0.084	0.28 (0.06-1.27)	0.100	0.27 (0.06-1.23)	0.091
Marital Status							
	Widowed	1.04 (0.20-5.57)	0.959				
	Married	0.52 (0.08-3.62)	0.515				
Education Level							
	Illiterate	0.75 (0.08-7.47)	0.808				
	Low	1.13 (0.12-10.93)	0.910				
	Average	0.90 (0.04-19.9)	0.950				
Household situation							
	Alone	0.90 (0.12-6.66)	0.923	1.41 (0.26-7.72)	0.686	1.65 (0.31-8.83)	0.552
	With Family	2.30 (0.40-13.4)	0.351	3.3 (0.74-15.2)	0.115	3.73 (0.83-16.8)	0.086
Financial situation							
	Unsatisfactory	0.59 (0.19-1.88)	0.373				
	Average	1.26 (0.56-2.86)	0.570				
Income							
	Individual	0.69 (0.33-1.46)	0.329			0.74 (0.44-1.24)	0.253
	Familiar	1.04 (0.70-1.5)	0.824				

The complete model presented a higher predictive value (62.5%) and specificity (60.8%). Model 1 had better sensitivity (77.7%), lower predictive value (48.1%) and specificity (42.9%). Model 2 presented worse sensitivity (69.4%), with a predictive value of 57.2% and specificity of 55% (Table 3). The choice for the best prediction model of frail long-lived individuals considered the parsimony rule and a higher index of sensitivity. Thus, we opted for the choice of Model 1.

**Table 3:** Comparison between predictive models for frailty in long-lived individuals. Curitiba, PR, Brazil, 2015.

	Complete Model	Model 1	Model 2
*p*-value	0.352	0.074	0.045
Prediction model	62.50%	48.10%	57.20%
Sensitivity	72.20%	77.70%	69.40%
Specificity	60.80%	42.90%	55%

## Discussion 

The condition of pre-frailty and frailty was significantly higher when compared to the *Cardiovascular Health Study* (CHS), which showed a prevalence of 46.6% of pre-frail older adults and 6.9% of frail older adults in a sample of 5,317 older/aged individuals, between 65 and 101 years old^2^. This result is due in part to the characteristic of the studied population, since they are older individuals aged 80 years or more. 

Results close to the present study are presented in cross-sectional surveys with 1,327 Spanish older adults (≥65 years), who identified 19.1% frail adults among the older adults in the group aged ≥75 years^19^; and the Frailty in Older Brazilian People Network (*FIBRA*) carried out in seven Brazilian cities revealed 19.7% as frail and 57.2% as pre-frail^20^ among the 512 long-lived individual participants. The high number of frail and pre-frail participants identified in this study similar to those long-lived individuals in the aforementioned investigations^19^ ^-^ ^20^ supports the relevance of preventive or therapeutic intervention actions in the care of long-lived individuals, with the objective of delaying or avoiding hospitalizations, falls and dependence, which are typical situations and events that occur due to physical frailty. 

Regarding the general characterization of the sample, the findings are similar to the results of national studies with long-lived individuals, which have indicated a greater number of women with a mean age of 84.4 years, widowed, with z low education level^21^ ^-^ ^22^, living with a partner and/or family members^21^ and receiving up to one minimum wage^22^. It is noticed that they are older women, with years marked only by the increase in life expectancy, who, nonetheless survive in undesirable physical and socioeconomic conditions, and for whom there is no specific care policy in force. 

We observed that long-lived women were twice as frail as long-lived individuals, however, a significant association was found between the female gender and frailty. It can be inferred that this result is due to the quantitative study participants, which represent a local reality in the South of Brazil. This result differs from others found in the literature which demonstrate such an association^2^ ^,^ ^19^ ^-^ ^20^. Among the contributory factors are physiological characteristics, unfavorable psychological and social conditions, stressors that interfere in the health status and contribute to an increase of accumulated deficits.

Among octogenarians (in their 80's) and nonagenarians (90's), the frequency of the syndrome did not increase with age, which can be explained by the categorization of the age groups which caused stratification of the aged population. However, univariate analysis revealed a significant association between age and frailty, similar to international and national studies^3^ ^-^ ^5^.

The significant increase in frail older adults in advanced ages suggests progressive condition of the syndrome, which is determined by physiological factors that can justify this relationship. In the perspective of the model proposed by North American authors^2^, the aging process predisposes individuals to develop physical frailty, and it can be related to changes and the decline of multiple systems, resulting from physiological mechanisms and pathological conditions^2^ which may be reflected in accumulated damage to the health and functionality of the aged individual^20^.

An analysis of marital status pointed to a higher proportion of frail widowed older adults, as expected for the age range of the study population and the predominantly female composition of the sample. A similar result was identified in other investigations^5^ ^,^ ^19^. However, unlike the longitudinal study with 1887 younger Italian aged adults, no significant association between widowhood and physical frailty was identified^23^. We highlight that widowhood can contribute to social and family isolation, and therefore lead to developing self-care deficit due to lack of encouragement from a partner.

It is noteworthy that more than half of the frail individuals had 1 to 4 incomplete years of study; however, this variable was not associated with the syndrome, corroborating a national study with community aged adults^5^. Nevertheless, a study conducted with 1,933 Mexican older adults at 65 years of age or older identified a higher probability of the syndrome in the aged with lower education levels (OR=2.51)^7^.

Despite developing countries having higher rates of illiteracy and lower education levels, a significant association between education level and the syndrome is found in developed countries such as Spain^19^ and Japan^4^. In this sense, the educational level can be considered a protection factor, since it provides individuals with better access to information and services, as well as financial resources and employment opportunities. 

The household situation variable had no significant association for the frail older adults, similar to a study developed with 203 aged people from Curitiba, whose objective was to investigate the association between frailty syndrome and sociodemographic and clinical characteristics of aged adult primary care users ^(^ ^24^. This result differs from those of other studies that observed this relationship among older adults who lived with family members^25^ and those living alone^2^ ^,^ ^4^ ^,^ ^7^. Social bonds and the support experienced can influence the health maintenance, promoting adaptive behavior in stress situations. 

Regarding the financial situation, no significant association between individual and family monthly income with the syndrome was found. However, some authors indicate that aged adults with unfavorable or insufficient income are more frail^7^ ^-^ ^8^. Socioeconomic conditions can trigger the physical frailty cycle in the aged as they hinder access to adequate food, health services, medicine and to practicing physical exercise, predisposing the individual to diseases and decreased functional capacity.

The predictive model of physical frailty chosen included variables that were associated with the syndrome: gender, age and household. Studies highlight advanced age and female gender as conditions strongly present in predictive models of frailty, both in the national^5^ and international context^2^ ^,^ ^25^.

The present study indicated greater chances of physical frailty in participants who lived with family members. This relationship was also observed (*p*=0.012)^25^ ^)^ similarly to a multicentric cross-sectional study conducted with 1,126 aged Turkish people investigating characteristics, prevalence and associated factors related to this syndrome in older adults. Higher odds of developing the syndrome in participants residing with family members can be attributed to the condition of long-lived individual's presenting some type of dependency (physical, financial or psychological), which may contribute to or accelerate the frailty process.

The cross-sectional design was a limiting factor in evaluating cause and effect relationships. Moreover, the sampling is representative of a local community, and therefore the results cannot be extrapolated to other territories. We suggest that further longitudinal, cohort, and population studies that allow for observing physical frailty levels in long-lived individuals and a deeper exploration of the relationship between the syndrome and sociodemographic variables be carried out.

Based on these results, we suggested that health professionals consider sociodemographic variables for screening physical frailty, seeking early identification of the syndrome. Thus, nursing care performed in basic care units can be targeted to specific groups of older adults and/or families (women, with advanced age, living with their family members) as an attempt to delay the frailty process and avoiding its negative outcomes.

## Conclusion 

Regarding the investigated sociodemographic variables, we can conclude that age significantly contributed to the frailty process in long-lived individual users of basic health care (units). The results show the influence of the aging process on the syndrome's occurrence, and it supports biological characteristics of the physical frailty phenotype proposed by North American authors.
